# The effects of atorvastatin on emotional processing, reward learning, verbal memory and inflammation in healthy volunteers: An experimental medicine study

**DOI:** 10.1177/02698811211060307

**Published:** 2021-12-06

**Authors:** Riccardo De Giorgi, Marieke Martens, Nicola Rizzo Pesci, Philip J Cowen, Catherine J Harmer

**Affiliations:** 1 Department of Psychiatry, Warneford Hospital, University of Oxford, Oxford, UK; 2 Warneford Hospital, Oxford Health NHS Foundation Trust, Oxford, UK

**Keywords:** Atorvastatin, emotional processing, reward learning, verbal memory, inflammation, experimental medicine, healthy volunteers, depression

## Abstract

**Background:**

Growing evidence from clinical trials and epidemiological studies suggests that statins can have clinically significant antidepressant effects, potentially related to anti-inflammatory action on several neurobiological structures. However, the underlying neuropsychological mechanisms of these effects remain unexplored.

**Aims:**

In this experimental medicine trial, we investigated the 7-day effects of the lipophilic statin, atorvastatin on a battery of neuropsychological tests and inflammation in healthy volunteers.

**Methods:**

Fifty healthy volunteers were randomised to either 7 days of atorvastatin 20 mg or placebo in a double-blind design. Participants were assessed with psychological questionnaires and a battery of well-validated behavioural tasks assessing emotional processing, which is sensitive to putative antidepressant effects, reward learning and verbal memory, as well as the inflammatory marker, C-reactive protein.

**Results:**

Compared to placebo, 7-day atorvastatin increased the recognition (*p* = 0.006), discriminability (*p* = 0.03) and misclassifications (*p* = 0.04) of fearful facial expression, independently from subjective states of mood and anxiety, and C-reactive protein levels. Otherwise, atorvastatin did not significantly affect any other psychological and behavioural measure, nor peripheral C-reactive protein.

**Conclusions:**

Our results reveal for the first time the early influence of atorvastatin on emotional cognition by increasing the processing of anxiety-related stimuli (i.e. increased recognition, discriminability and misclassifications of fearful facial expression) in healthy volunteers, in the absence of more general effects on negative affective bias. Further studies exploring the effects of statins in depressed patients, especially with raised inflammatory markers, may clarify this finding and inform future clinical trials.

## Introduction

Statins are a class of anti-cholesterolemic drugs widely used for the prevention and treatment of cardiovascular, cerebrovascular and metabolic disorders (Sizar et al., 2021). Historically, their primary mechanism of action was recognised as the inhibition of the rate-limiting liver enzyme, 3-hydroxy-3-methylglutaryl-coenzyme A (HMG-CoA) reductase (Endo et al., 1976), with consequent decline of blood cholesterol levels (Law et al., 2003). However, statins also have pleiotropic effects that, either directly or indirectly, affect many neurobiological (neurotransmission, neurogenesis, oxidative stress and excitotoxicity), cardiometabolic and immune systems that have been implicated in the aetiopathogenesis of depression (Köhler-Forsberg et al., 2020). Indeed, the anti-inflammatory effects of statins are of particular interest in this respect because of the postulated role of inflammation in the pathophysiology of depression (Miller et al., 2017).

Both observational, epidemiological studies (Parsaik et al., 2014) and randomised clinical trials (Salagre et al., 2016) have suggested that statins may have clinically meaningful antidepressant effects (De Giorgi et al., 2021a). For example, in a recent within-subject cohort study of over one million statin-treated patients, Molero et al. (2020) reported lower rates of depression diagnoses during periods when patients were receiving statins relative to when they were not. A previous cohort investigation found that patients taking selective serotonin reuptake inhibitors (SSRIs) in combination with statins had lower rates of psychiatric hospital contacts for depression than patients taking SSRIs alone (Köhler et al., 2016). A previous meta-analysis of a small number of placebo-controlled, randomised trials found that the combination of statins SSRIs produced a greater decrease in depression ratings than SSRIs given alone (Salagre et al., 2016). However, a more recent study of rosuvastatin in young people with depression (only some of whom were also taking antidepressant medication) showed a numerical but not statistically significant benefit of statin treatment over placebo on the primary end-point of difference in depressive scores (Berk et al., 2020).

Despite these promising antidepressant effects of statins in different kinds of clinical investigation, as far as we are aware, there are no experimental studies of the effects of statins on emotional processing. We have previously shown that a variety of pharmacological agents with established antidepressant effects produce rapid positive biases in tasks of emotional processing in both healthy participants and patients with depression (Harmer et al., 2017; Pringle et al., 2011). Measures of emotional processing therefore have the potential to detect the putative antidepressant efficacy of novel compounds (Harmer et al., 2011, 2017). Interestingly, the same tasks have been able to detect negative effects on emotional processing produced by drugs of known depressogenic potential such as the cannabis receptor antagonist, rimonabant (Horder et al. 2012). Similarly, induction of inflammation in healthy participants, a procedure known to cause depressive symptoms, produces negative emotional biases (Cooper et al., 2018), deficit in reward-seeking behaviour (Felger and Treadway, 2017), and overall cognitive impairment (Paine et al., 2015).

The primary aim of this study was to assess the effect of short-term (7 days) treatment with the lipophilic statin, atorvastatin, upon a battery of emotional processing tasks previously shown to be sensitive to the early effects of standard antidepressants such as the SSRI, citalopram. Secondarily, we investigated the effects of statin treatment on reward learning and verbal memory. As noted above, the antidepressant effects of statins have been linked to their anti-inflammatory properties; we therefore also explored the effect of this period of statin use on plasma levels of the inflammatory marker high-sensitivity C-reactive protein (hs-CRP) in relation to potential changes in the behavioural tasks. To our knowledge, no previous experimental medicine trials have studied the effects of statins on behavioural tasks assessing emotional processing, reward learning and verbal memory. However, in view of previous evidence suggesting that statins may exert antidepressant effects, we tentatively predicted that 7-day atorvastatin would have positive effects on these measures.

## Methods

The protocol for this double-blind, parallel groups, randomised (gender-stratified), placebo-controlled, experimental medicine trial was approved by the University of Oxford Central University Research Ethics Committee (MS-IDREC R61966/RE001) and registered on Clinicaltrials.gov (NCT03966859).

## Participants

Fifty healthy volunteers (males and females, aged 18–50 years, body mass index: 18–30) were recruited for this study and provided full written informed consent. Participants were screened to be free of current or past mental disorders via the structured clinical interview for Diagnostic and statistical manual of mental disorders (5th ed.; DSM-5) (First et al., 2016) and identified as ‘healthy’ on the basis of the latter as well as their reported psychiatric, medical and drug history (no regularly prescribed medications, no psychotropic drugs over the last 3 months). Women who were pregnant, breastfeeding or of child-bearing potential not using appropriate contraceptive measures were excluded. Participants who withdrew from the study were replaced.

Sample size calculation was performed with software G*Power v3.1.9.6 (Faul et al., 2007) and based on the primary outcome of accuracy at recognising emotional facial expressions derived from a previous study of antidepressants in healthy volunteers (Harmer et al., 2004), where a *N* = 38 would give 90% power to detect changes of a comparable magnitude (citalopram vs placebo effect size *F* = 0.5, see Supplemental Material, S1).

## Study procedures

Potential participants were screened according to pre-specified inclusion and exclusion criteria (see above and registered protocol NCT03966859). If eligible, they were asked to complete a battery of questionnaires including the Beck Depression Inventory (BDI, Beck et al., 1961), the Eysenck Personality Questionnaire (EPQ, Eysenck et al., 1985), the Positive and Negative Affect Schedule (PANAS, Watson et al., 1988), the Snaith–Hamilton Pleasure Scale (SHAPS, Snaith et al., 1995), a side-effects questionnaire (nausea, dizziness, dry mouth, headache, alertness and agitation scored from 0 to 3), the State-Trait Anxiety Inventory (STAI, Spielberger et al., 1970) and the Bond-Lader Visual Analogue Scales (BL-VAS, Bond and Lader, 1974) at baseline. Also, a first blood sample for measuring hs-CRP was collected.

Enrolled participants were then randomised according to a randomisation code drawn up by a researcher uninvolved in the study using an online randomisation tool (https://www.sealedenvelope.com/simple-randomiser/v1/lists) to either atorvastatin 20 mg or sucrose placebo, encapsulated in identical opaque white capsules, taken orally once daily for 7 days. A 20-mg atorvastatin dose was selected as this is considered a safe and commonly used dosage for cardiometabolic disorders, and it has been employed in previous clinical trials in depression (Abbasi et al., 2015; Haghighi et al., 2014). To monitor compliance, participants were asked to report their adherence and to return their capsules’ bottles on the day of testing.

After 7 days of atorvastatin or placebo administration, participants attended a testing visit involving the completion of further questionnaires (PANAS, side-effects questionnaire, STAI-state, BL-VAS), a second sample for hs-CRP and the administration of the behavioural tasks described below.

## Behavioural tasks

The behavioural tasks included the emotional test battery (ETB), the probabilistic instrumental learning task (PILT) and the auditory-verbal learning task (AVLT).

## ETB

The ETB assesses emotional processing (‘hot cognition’) with five well-validated computerised cognitive tasks: the facial expression recognition task (FERT), the Emotional Categorisation Task (ECAT), the Emotional Recall Task (EREC), the Emotional Recognition Memory Task (EMEM) and the Faces Dot-Probe Task (FDOT), which have previously been described in full (Harmer et al., 2009).

The FERT involved various facial expressions showing six emotions: anger, disgust, fear, happy, sad, surprise and a neutral one, selected and modified from the Karolinska Directed Emotional Faces (KDEF) set (Lundqvist et al., 1998) each at a range of intensity levels (Young et al., 1997). The facial stimuli were randomly presented on the computer screen for 500 ms and replaced by a blank screen. Participants were probed to identify as quickly and as accurately as possible the emotion of the face displayed in front of them and gave their responses by pressing a labelled key on the keyboard. Accuracy to identify the correct emotion was the primary outcome measure for this study. A signal detection analysis was used to assess discriminability (*d′*, perceptual choice, a measure of sensitivity), and response bias (β, decisional choice, a measure of conservativeness) (Grier, 1971). A further analysis was performed using the change in hs-CRP from the baseline to the testing visit as a covariate to investigate whether potential differences between groups could be explained by change in inflammatory status. Mean reaction times and misclassifications for each emotion were also recorded.

The ECAT asked participants to indicate as quickly and as accurately as possible by pressing a labelled key on the keyboard whether they would like or dislike to be referred to as one of a series of 60 positively (e.g. cheerful) or negatively valenced (e.g. hostile) words (Anderson, 1968) presented on the computer screen for 500 ms. These words were matched in terms of word length, ratings of frequency, and meaningfulness. Accuracy and mean reaction times for positive compared to negative words were recorded. The EREC was a surprise free-recall task during which participants were required to recall and write down as many self-referent words from the ECAT as possible in 4 min. Correctly (hits) and falsely recalled (false alarms) words were recorded. The EMEM included 60 self-referent words from the ECAT, alongside 60 matched distractors (30 positive, 30 negative) presented on the computer screen for 500 ms. Participants were required to recognise them as previously seen (familiar) or unseen (novel) in the ECAT by pressing a labelled key on the keyboard. Accuracy, reaction times and false alarms (incorrectly attributing a previously unseen word to the ECAT) were recorded.

In the FDOT, two faces (either an emotional (fearful or happy) or a neutral face) were shown at the top and at the bottom of the computer screen and then replaced by a pair of dots, to which the participant had to respond by indicating whether the dots are vertically or horizontally aligned by pressing a labelled key on the keyboard. On half the total 192 trials, the faces were presented very briefly and immediately switched to a muddled face mask. Attentional vigilance scores were calculated by subtracting the mean reaction time from trials when probes appeared in the same position as the emotional face (congruent trials) from trials when probes appeared in the opposite position to the emotional face (incongruent trials).

## PILT

The PILT assesses probabilistic instrumental learning to reward/loss. This is a modified version of that described in Pessiglione et al. (2006) and has previously been described in full (Walsh et al., 2018). The task stimuli presented on the computer screen consisted of two pairs of symbols displayed for 4000 ms with one pair associated with win outcomes (win £0.20 or no change) and the other associated with loss outcomes (lose £0.20 or no change). Each symbol in the pair corresponded to reciprocal probabilities (0.7 or 0.3) of the associated outcomes occurring and was randomly positioned either to the left or the right of a central fixation cross. Participants began the task with £1.50 and performed 60 independent trials (30 win trials and 30 loss trials), for two runs, receiving outcome feedback (win or lose) after each trial. To maximise their winnings, participants should use the outcome feedback to gradually learn the symbol-outcome associations over time, such that they consistently chose the symbol with the high-probability win and avoided the symbol with the high-probability loss. Outcome measures, averaged across the two runs, included end total amount, amount won, amount lost and number of choice switches (proportion of trials in which the chosen symbol was different to the symbol chosen in the previous trial within the same condition, therefore a measure of confidence such that the lower the proportion of switch trials, the greater the confidence the participant is thought to have had in their choices).

## AVLT

The AVLT assesses declarative verbal memory (‘cold cognition’) (Rey, 1964), through a list of 15 nouns (List A) that participants were asked to recall and repeat to the researcher. After five repetitions of free-recall, a second ‘interference’ list (List B) was presented and assessed in the same manner. After this trial, participants were immediately asked to recall the words from List A. After a 20-min delay, participants were asked to again recall the words from List A. Overall, correct words, intrusions and repetitions were measured. After this ‘delayed recall’ task, a list of 50 words was presented containing all of the words from Lists A and B as well as distracting words; participants were then asked to indicate if the word belonged to List A or not. Correctly (hits) and falsely recalled (false alarms) words were recorded.

## Statistical analysis

IBM SPSS v27 statistical software (IBM, 2020) was used to analyse the data. Data distributions were visually checked using boxplots and corrected for extreme outliers (i.e. data values which lie more than 3 times the interquartile range below the first quartile or above the third quartile were excluded). Demographics, clinical characteristics and baseline questionnaires were reported descriptively. Repeated questionnaires (PANAS, side-effects questionnaire, STAI-state, BL-VAS) and hs-CRP were analysed using repeated-measures analysis of variance (ANOVA) with group (atorvastatin vs placebo) as the between-subject factor and time (baseline vs testing visit) as the within-subject factor. Behavioural tasks’ results were analysed using repeated-measures ANOVA with group as the between-subject factor and emotion/valence as the within-subject factor. Any significant interaction was followed up using simple main effect analyses. When assumptions of equality of variances were not fulfilled, the Greenhouse–Geisser procedure was used to correct the degrees of freedom. Partial eta squared (η^2^) was reported for the main significant comparisons as a measure of effect size (η^2^ = 0.01, small effect; η^2^ = 0.06, medium effect; η^2^ = 0.14, large effect).

## Results

### Baseline measures

The sample’s demographic, clinical and baseline questionnaires (BDI, EPQ, SHAPS, STAI-trait) data are detailed in Table 1. By the end of the study, 22 participants were randomised to atorvastatin (11 females) and 28 (14 females) to placebo.

**Table 1.** Sample’s demographics, clinical characteristics and baseline questionnaires.

	Atorvastatin	Placebo
Sample size	22	28
Gender	11 F/11 M	14 F/14 M
Age	24.8 (6.0)	27.6 (7.3)
English as first language	13	17
Education		
High school/college	1	2
Undergraduate	14	14
Postgraduate	7	12
Family history of mental disorder	9	12
Smoke/day	0.1 (0.2)	0.3 (1.0)
Alcohol units per week	7.0 (6.3)	5.9 (5.3)
Caffeinated drinks per day	1.8 (1.2)	1.5 (1.2)
BMI	21.7 (2.4)	22.9 (3.1)
BDI	1 (1.4)	1.4 (1.8)
EPQ		
Neuroticism/stability	5.2 (3.5)	6.0 (4.8)
Psychoticism/socialisation	3.0 (1.9)	2.3 (2.0)
Extroversion/introversion	14.3 (3.6)	14.7 (3.8)
Lie/social desirability	8.9 (4.9)	9.6 (4.3)
SHAPS	0.6 (1.5)	1.0 (1.9)
STAI-t	30.9 (5.5)	28.5 (6.7)

BDI: Beck Depression Inventory; BMI: body mass index; EPQ: Eysenck Personality Questionnaire; SHAPS: Snaith–Hamilton Pleasure Scale; STAI-t: State-Trait Anxiety Inventory-Trait.

Values represent means (standard deviations).

### Testing visit measures

The sample’s questionnaires (PANAS, STAI-state, side-effects questionnaire) and hs-CRP data, measured both at baseline and testing visit, are detailed in Table 2. For the PANAS-positive, there was a main effect of time of visit (*F*_1,48_ = 6.28, *p* = 0.02) due to higher scores in both groups at the testing visit in comparison to the baseline visit, but no group-time interaction (*F* < 0.91, *p* > 0.20). The side-effects questionnaire showed a significant group-time interaction (*F*_1,48_ = 4.33, *p* = 0.04) due to higher scores at the testing visit in comparison to the baseline visit for the placebo group and the opposite for the atorvastatin group. For the hs-CRP, there was a main effect of time of visit (*F*_1,47_ = 5.91, *p* = 0.02) due to lower scores in both groups at the testing visit in comparison to the baseline visit, but no group-time interaction (*F* < 0.11, *p* > 0.20). The PANAS-negative and STAI-state did not show any main effect of time of visit or group-time interaction (*F* < 1.78, *p* > 0.20).

**Table 2.** Sample’s repeated questionnaires and hs-CRP.

	Atorvastatin		Placebo		*p* visit	*p* group × visit
	Screening visit	Research visit	Screening visit	Research visit		
PANAS-positive	31.86 (5.67)	33.23 (6.23)	33.32 (7.93)	36.36 (7.11)	0.02*	>0.20
PANAS-negative	12.14 (2.75)	13.18 (3.03)	12.14 (2.21)	12.11 (2.69)	>0.20	>0.20
Side-effects	2.14 (1.13)	1.68 (1.25)	2.18 (1.72)	2.57 (1.67)	>0.20	0.04*
STAI-s	32.27 (5.53)	32.64 (6.73)	31.64 (8.91)	30.00 (6.79)	>0.20	>0.20
hs-CRP	1.44 (1.41)	1.04 (1.30)	1.55 (1.75)	1.02 (1.05)	0.02*	>0.20

hs-CRP: high-sensitivity C-reactive protein; PANAS: Positive and Negative Affect Schedule; STAI-s: State-Trait Anxiety Inventory-state.

Values represent means (standard deviations).

* A statistically significant difference between the atorvastatin and placebo groups.

For the BL-VAS (descriptive statistics in Supplemental Material, S2), the items ‘clumsy- well coordinated’ and ‘antagonistic-friendly’ showed a main effect of time of visit (respectively, *F*_1,48_ = 6.09, *p* = 0.02 and *F*_1,48_ = 5.09, *p* = 0.03) due to lower scores in both groups at the testing visit in comparison to the baseline visit, but no group-time interaction (*F* < 0.21, *p* > 0.20). The item ‘sad-happy’ showed a significant group-time interaction (*F*_1,48_ = 6.33, *p* = 0.01) due to higher scores at the testing visit in comparison to the baseline visit for the placebo group (scored more towards happiness) and the opposite for the atorvastatin group (scored more towards sad). All the other items on this scale did not show any main effect of time of visit or group-time interaction (*F* < 0.92, *p* > 0.20).

### Behavioural tasks

All descriptive statistics are reported in Supplemental Material, S3. No participant was excluded for being an extreme outlier.

### ETB results

**Figure 1.**
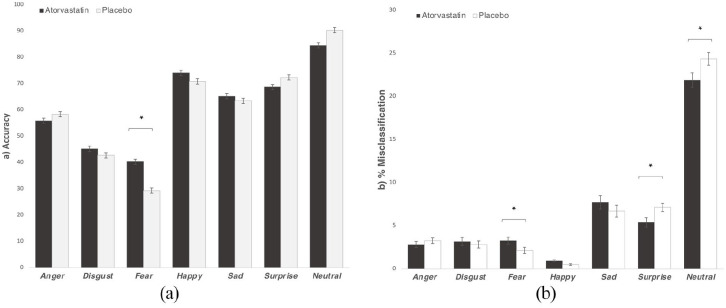
Effect of atorvastatin on facial expression recognition (FERT). Results for accuracy (a) and misclassifications percentage (b) for all seven emotions are shown. Values are means ± standard error of the mean bars. *A statistically significant difference between the atorvastatin (dark) and placebo (white) groups.

*FERT*. For facial expression recognition (Figure 1), we found a significant group-emotion interaction for total accuracy (*F*_6,288_ = 3.46, η^2^ = 0.07, *p* = 0.01) and sensitivity index (*d’ F*_6,288_ = 2.61, η^2^ = 0.06, *p* = 0.04), but no group-emotion interaction for response bias (β *F*_6,288_ = 1.20, *p* > 0.30); pairwise comparisons revealed that participants receiving atorvastatin were more accurate at recognising fear than those on placebo (*F*_1,48_ = 8.64, *p* = 0.006; *d’ F*_1,48_ = 2.62, *p* = 0.03), but not other emotions (*F* < 2.9, *p* > 0.10). These results remained consistent when hs-CRP was used as a covariate. Because of the finding of a group-time interaction for the ‘sad-happy’ item on the BL-VAS, this was also analysed as a covariate, but again outcome scores did not change. Moreover, there was a significant group-emotion interaction for total misclassifications (*F*_6,288_ = 3.25, η^2^ = 0.06, *p* = 0.02), with follow-up pairwise comparisons showing that the atorvastatin group were more likely to falsely classify faces as fearful (*F*_1,48_ = 4.38, *p* = 0.04) and less likely to misclassify faces as surprised (*F*_1,48_ = 5.91, *p* = 0.02) or neutral (*F*_1,48_ = 4.88, *p* = 0.03). When positive (happy, surprise) versus negative (fear, disgust, anger, sadness) emotions were compared, no effect for group or group-emotion interaction was identified (*F* < 2.81, *p* > 0.10). Likewise, reaction times did not show any effect for group or group-emotion interaction (*F* < 0.78, *p* > 0.55).

*Other ETB tasks.* No significant group or interaction effects were observed for all measures of assessment of word valence (ECAT *F* < 0.45, *p* > 0.40), recall (EREC) and recognition (EMEM) of positive or negatively valenced words (*F* < 1.61, *p* > 0.20), and attentional vigilance towards happy or fearful faces (FDOT *F* < 1.77, *p* > 0.20).

### Probabilistic instrumental learning task results

For reward as measured by the PILT, an interaction effect was observed for the total number of choice switches (*F*_1,48_ = 4.92, *p* = 0.03); however, post hoc analyses showed no significant differences between the atorvastatin and placebo groups for the losses or wins conditions (*p* > 0.70). Also, no significant differences were identified for all other outcomes (*F* < 1.42, *p* > 0.20).

### AVLT results

For verbal memory as measured by the AVLT, no significant differences between the atorvastatin and placebo groups were identified for any of the outcomes measured (*F* < 0.82, *p* > 0.20).

## Discussion

To our knowledge, this is the first study to investigate the psychological effects of a statin in healthy volunteers with a battery of emotional processing tasks under laboratory conditions.

Overall, we observed that 7-day atorvastatin treatment, compared to placebo, led to increased recognition, sensitivity and misclassifications for fearful facial expressions, but not for other emotions, independently from subjective states of mood and anxiety, and C-reactive protein levels. We did not identify any further significant changes for other tasks measuring emotional processing, nor for those assessing reward learning and verbal memory. In addition to our primary analysis on accuracy in recognising emotional facial expressions, we conducted several other analyses that were not corrected for multiple comparisons because of our relatively small sample size, which is a limitation to our study. However, the majority of these additional analyses did not show any statistically significant difference between the atorvastatin and the placebo group, which mitigates the potential reporting of false positives.

The finding that atorvastatin can affect emotional processing is of particular interest, since changes in cognitive affective bias in the early stages of drug treatment have been shown to be predictive of antidepressant efficacy or alternatively adverse effects on mood (Harmer et al., 2011, 2017; Horder et al. 2012). More specifically, we found convincing evidence that 7-day atorvastatin administration compared to placebo increases the identification of fearful facial expressions in healthy volunteers, as highlighted by greater accuracy and discriminability for recognising fear as well as a higher number of fear misclassifications. The lack of any change in response bias suggests that the effect concerns sensitivity to fearful stimuli rather than a decisional choice bias. Although a negative effect of atorvastatin was observed on the BL-VAS item ‘sad-happy’, our results remained consistent when this element was introduced as a covariate; otherwise, no significant changes in subjective states of mood and anxiety were identified. These results appear in contrast to our initial, cautious prediction that 7-day atorvastatin treatment would be associated with a positive effect on emotional processing, and therefore deserve further discussion.

Increased fear recognition may reflect an augmented processing of threatening cues and a worsening of negative bias, both of which have been associated with acute SSRI administration (Browning et al., 2007; Godlewska and Harmer, 2021). Also, anxiety states have been linked to increased recognition of fearful facial expressions (Mogg and Bradley, 2002) and administration of anxiolytic drugs such as the benzodiazepine and diazepam has been shown to specifically decrease the recognition of fear (Zangara et al., 2002). On these bases, one could argue that atorvastatin may play a role in worsening anxiety symptoms. Indeed, a recent animal study showed that high doses of simvastatin or rosuvastatin caused increased anxiety in rats (Okudan and Belviranli, 2020). However, this appears in contrast with clinical research findings. A randomised controlled trial found no significant difference in anxiety scores between rosuvastatin and placebo given in addition to treatment as usual (antidepressant, psychological therapy) for 12 weeks to a young sample of patients with depression (Berk et al., 2020). More significantly, a large cohort study on a Swedish nationwide register of statin users followed up for 8 years showed no effect for all statins on the risk of anxiety disorders (Molero et al., 2020), whereas a previous observational study on patients with coronary artery disease even reported a small improvement (odds ratio = 0.69, 95% confidence interval = 0.47–0.99) in anxiety symptoms among 4-year statin users (Young-Xu et al., 2003). It should be noted that all the clinical studies involved a much longer period of statin use compared to our 7-day administration; moreover, none of these studies specifically used atorvastatin. The interpretation of our main finding, namely that 7 days of atorvastatin increases fear processing, might therefore be more complex and requires further contextualisation.

Changes in the recognition of fearful expressions are most frequently reported in studies exploring the effects on emotional processing of commonly prescribed antidepressants (Harmer and Cowen, 2013). For example, the administration to healthy participants of a single dose of the SSRI citalopram, respectively, intravenously (Harmer et al., 2003) and orally (Browning et al., 2007), led to higher detection of facial expressions of fear. A similar effect has been seen with acute administration of serotonin precursor, tryptophan (Attenburrow et al., 2003). However, when citalopram was given for 7 days, a reduction in fear recognition was observed (Harmer et al., 2004). These results appear to parallel clinical experience with SSRIs: an early exacerbation of anxiety and agitation usually followed by anxiolytic effects on prolonged treatment (Harmer et al., 2011) – an effect that has been similarly observed in animal models of anxiety (Burghardt et al., 2004). This reversal of action seems to involve a desensitisation of 5HT_2c_ receptors (Deakin and Graeff, 1991); in line with this, the 5HT_2a/c_ receptor blocker mirtazapine immediately reduced fear recognition (Arnone et al., 2009).

It is tempting to match our finding that 7-day atorvastatin heightens threat processing in a way that is similar to the acute effect of SSRIs (Browning et al., 2007; Burghardt et al., 2004; Harmer et al., 2003). Then, consistently with clinical studies, continued treatment with statins might lead to a paradoxical improvement in anxiety (Berk et al., 2020; Molero et al., 2020; Young-Xu et al., 2003) and indeed depressive symptoms (Parsaik et al., 2014; Salagre et al., 2016). The neuropsychological and neurobiological underpinnings for such delayed effect appearing even longer than with SSRIs (Harmer et al., 2017) remain unclear. Although some of the peripheral, especially anti-inflammatory, actions of statins seem relatively fast (i.e. <7 days) (Macin et al., 2005), the complex downstream effects on the central nervous system (CNS) and its dedicated immune system, and thus on anxiety and depressive symptoms, may be slower. Additional experimental medicine trials examining the effects of long-term administration of statins on emotional processing, possibly complemented by the use of more sensitive techniques such as functional Magnetic Resonance Imaging (fMRI), may help clarify this issue.

The most established effects of statins on cholesterol and inflammation (Jain and Ridker, 2005) also warrant further consideration. We did not observe any significant change in hs-CRP due to atorvastatin as compared to placebo, and its levels did not seem to affect measures of emotional processing. However, this exploratory analysis should be interpreted with caution since hs-CRP levels were not generally elevated (see Table 2) for both groups in this sample of healthy volunteers. A mediating effect of CRP on emotional processing could still become apparent in a study conducted on depressed participants with elevated baseline hs-CRP. Moreover, we did not measure blood cholesterol levels, and this is a limitation that other studies may want to explore. Another study from our group has recently shown that a single dose of the antibiotic minocycline, which possesses putative antidepressant mechanisms, acutely decreases fear misclassifications on the ETB while increasing cholesterol and decreasing CRP levels in healthy volunteers (Chan et al., 2020).

Intriguingly, a previous clinical trial of the anti-inflammatory drug infliximab in a depressed sample did not identify any effect on mood scores until participants were stratified according to their serum CRP: those with CRP >5.0 mg/L improved during infliximab treatment, whereas those with CRP below that threshold even showed a deterioration of their depressive symptoms (Raison et al., 2013). On the basis of several animal studies, it has been hypothesised that a degree of ‘healthy inflammation’ is necessary to maintain homeostatic processes of neuronal integrity, such as neuroplasticity and neurogenesis, that are involved in the pathophysiology of depression (Miller et al., 2017); in other words, any undue curbing of natural inflammatory processes in the CNS might be as detrimental as an excessive amount of neuroinflammation. It should be noted that our sample of healthy and mostly young volunteers, perhaps unsurprisingly, presented with overall quite low (mean = 1.58 mg/L) levels of hs-CRP across the two visits. Moreover, there is evidence suggesting that younger depressed patients administered a statin may encounter less benefit on depressive symptoms (Berk et al., 2020), whereas a step-wise reduction in the risk of emerging depressive episodes with increasing age was observed for statin users (Redlich et al., 2014). Although peripheral CRP and young age may not necessarily reflect baseline inflammation in the CNS, this raises the possibility that atorvastatin produced a worsening negative bias in our young participants because it impaired physiological homeostatic processes involved in emotional processing. Furthermore, the use of atorvastatin doses other than the 20 mg employed in this study could variably affect such homeostasis, and therefore lead to more positive or negative effects on emotional processing. Atorvastatin is safely administered to patients with cardiometabolic conditions in dose ranges between 10 and 80 mg according to guidelines (Chou et al., 2016). Therefore, further studies investigating these other dosages may help clarify whether there is any dose–response relationship between atorvastatin use, anti-inflammatory effects and emotional processing.

It has been advocated that future clinical trials in depression should follow precision medicine principles by selecting a priori a subset of patients with elevated inflammatory markers, such as CRP, who most likely would respond to anti-inflammatory drugs (Chamberlain et al., 2019). The same strategy could be applied to translational research: the next experimental medicine studies of statins ought to focus on samples of patients with depression or at-risk of depressive episodes, possibly pre-selected for high CRP, to elucidate whether our results are replicated or indeed reversed in these specific clinical samples. Furthermore, inflammatory processes and lipid metabolism physiologically undergo complex interactions (Hudgins et al., 2003; Van Diepen et al., 2013) that are liable to be further complicated by the distinct activity of statins on these two systems. Lipids are crucial to brain structure and function, and there is evidence that changes in CNS cholesterol, phospholipids and sphingolipids are associated with psychiatric disorders (Schneider et al., 2017) and, in animals, they seem to interact with antidepressant drugs (Gulbins et al., 2013; Lee et al., 2009). Thus, the emerging field of lipidomics in psychiatric disorders and especially in depression (Walther et al., 2018) suggests that further characterisation of the participants’ lipid profile might need to be taken into account when interpreting the effect of statins on depressed patients.

On a related note, although most statins may share similar effects, their ability to cross the blood-brain barrier varies according to their lipophilicity (McFarland et al., 2014), and might therefore influence the local expression of such effects in the CNS. For example, a previous observational study showed that while simvastatin seemed to reduce the risk of a new diagnosis of depression (odds ratio = 0.93, 95% confidence interval = 0.89–0.97), atorvastatin showed the opposite effect (odds ratio = 1.11, 95% confidence interval = 1.01–1.22) (Redlich et al., 2014). In this study, we used a relatively low dose (20 mg) of atorvastatin, which has a reasonably high lipophilic index though lower than simvastatin and lovastatin (Corsini et al., 1999). However, it is possible that the negative effect on emotional processing seen in this study may be specific to atorvastatin and that other statins could behave differently.

Notably, observational evidence indicates that simvastatin had the most beneficial effect in reducing the odds of depression (odds ratio = 0.93, 95% confidence interval = 0.89–0.97) among all statins (Molero et al., 2020) and compared to atorvastatin (Abbasi et al., 2015). A recent exploratory network meta-analysis from our group showed a similar trend in randomised controlled trials (De Giorgi et al., 2021b), though this result has not been replicated in another study (De Giorgi et al., 2021c; Lee et al., 2021). Conversely, several recent trials using large-molecule anti-inflammatory agents incapable of penetrating the brain parenchyma have reported little overall effects on depressive scores (Husain et al., 2020; McIntyre et al., 2019; Salvadore et al., 2018). Since central inflammation seems to be as important as peripheral inflammation in depression (Enache et al., 2019), drugs which are capable of regulating inflammatory processes within the CNS might be more likely to express a therapeutic effect in depressed patients. Hence, clinical trials of statins in depressed patients too ought to consider using a statin with high brain penetration such as simvastatin – as seems to be the case in currently running clinical studies (Husain et al., 2019; Otte et al., 2020).

Impaired reward processing, related to anomalies in dopamine neurotransmission, is widely regarded as the foundation of the anhedonia that often characterises an inflammatory phenotype of depression (Miller et al., 2017). In our study, we used the PILT to verify whether atorvastatin had any effects on reward learning, but found no consistent effects. In view of the apparent close relationship between inflammatory processes and the functioning of reward circuitry, it is again possible that we failed to detect significant effects of atorvastatin in this task because our sample did not have baseline high inflammation, or the dose and type of statin we employed could not express its full potential in the CNS. Also, a longer period of statin administration could have gradually affected downstream effects on the dopaminergic system that may have resulted in changes on the PILT. For example, the norepinephrine-dopamine reuptake inhibitor (NDRI) bupropion was found to cause an initial worsening of reward processing at 2 weeks in depressed participants which was reversed and normalised only after 6 weeks of treatment (Walsh et al., 2018). It should also be noted that this study was powered on the primary outcome of accuracy at recognising emotional facial expressions; therefore, it may have been underpowered (i.e. increased false negatives) to capture changes in reward learning. The same caveats could be applied to the apparent lack of effects of atorvastatin on verbal memory, although we did not necessarily expect to see any difference on the AVLT since reports on the effects of statin on ‘cold’ cognitive functions have been inconsistent (Schultz et al., 2018).

Finally, although the short duration of treatment may have not captured long-term side effects, we could add to the extensive evidence confirming the safety profile of statins (Collins et al., 2016) as evidenced by scores lower than placebo on the side-effects questionnaire.

In summary, our study is the first to identify the early effects of atorvastatin on emotional processing in healthy volunteers, in the absence of any important changes in subjective state. Although the lack of the potentially confounding effects of depressive and anxiety symptoms is suitable for a mechanistic study as such, the clinical relevance of our findings cannot currently be specified. We recommend that future experimental medicine trials should explore the effects of statins in a depressed population, possibly with elevated markers of baseline inflammation, to determine whether early changes in emotional processing might predict subsequent beneficial effects on clinical symptoms of depression and anxiety.

## Supplemental Material

sj-docx-1-jop-10.1177_02698811211060307 – Supplemental material for The effects of atorvastatin on emotional processing, reward learning, verbal memory and inflammation in healthy volunteers: An experimental medicine study Supplemental material, sj-docx-1-jop-10.1177_02698811211060307 for The effects of atorvastatin on emotional processing, reward learning, verbal memory and inflammation in healthy volunteers: An experimental medicine study by Riccardo De Giorgi, Marieke Martens, Nicola Rizzo Pesci, Philip J Cowen and Catherine J Harmer in Journal of Psychopharmacology

## References

[bibr1-02698811211060307] Abbasi SH Mohammadinejad P Shahmansouri N , et al. (2015) Simvastatin versus atorvastatin for improving mild to moderate depression in post-coronary artery bypass graft patients: A double-blind, placebo-controlled, randomized trial. Journal of Affective Disorders 183: 149–155. 26005776 10.1016/j.jad.2015.04.049

[bibr2-02698811211060307] Anderson NH (1968) Likableness ratings of 555 personality-trait words. Journal of Personality and Social Psychology 9: 272–279. 5666976 10.1037/h0025907

[bibr3-02698811211060307] Arnone D Horder J Cowen PJ , et al. (2009) Early effects of mirtazapine on emotional processing. Psychopharmacology 203(4): 685–691. 19031070 10.1007/s00213-008-1410-6

[bibr4-02698811211060307] Attenburrow MJ Williams C Odontiadis J , et al. (2003) Acute administration of nutritionally sourced tryptophan increases fear recognition. Psychopharmacology 169(1): 104–107. 12719963 10.1007/s00213-003-1479-x

[bibr5-02698811211060307] Beck AT Ward CH Mendelson M , et al. (1961) An inventory for measuring depression. Archives of General Psychiatry 4: 561–571. 13688369 10.1001/archpsyc.1961.01710120031004

[bibr6-02698811211060307] Berk M Mohebbi M Dean OM , et al. (2020) Youth depression alleviation with anti-inflammatory agents (YoDA-A): A randomised clinical trial of rosuvastatin and aspirin. BMC Medicine 18(1): 16. 31948461 10.1186/s12916-019-1475-6 PMC6966789

[bibr7-02698811211060307] Bond A Lader M (1974) The use of analogue scales in rating subjective feelings. British Journal of Medical Psychology 47: 211–218.

[bibr8-02698811211060307] Browning M Reid C Cowen PJ , et al. (2007) A single dose of citalopram increases fear recognition in healthy subjects. Journal of Psychopharmacology 21(7): 684–690. 17259206 10.1177/0269881106074062

[bibr9-02698811211060307] Burghardt NS Sullivan GM McEwen BS , et al. (2004) The selective serotonin reuptake inhibitor citalopram increases fear after acute treatment but reduces fear with chronic treatment: A comparison with tianeptine. Biological Psychiatry 55(12): 1171–1178. 15184036 10.1016/j.biopsych.2004.02.029

[bibr10-02698811211060307] Chamberlain SR Cavanagh J de Boer P , et al. (2019) Treatment-resistant depression and peripheral C-reactive protein. The British Journal of Psychiatry 214(1): 11–19. 29764522 10.1192/bjp.2018.66 PMC6124647

[bibr11-02698811211060307] Chan SY Capitão L Probert F , et al. (2020) A single administration of the antibiotic, minocycline, reduces fear processing and improves implicit learning in healthy volunteers: Analysis of the serum metabolome. Translational Psychiatry 10(1): 148. 32404908 10.1038/s41398-020-0818-6 PMC7220900

[bibr12-02698811211060307] Chou R Dana T Blazina I , et al. (2016) Statin Use for the Prevention of Cardiovascular Disease in Adults: A Systematic Review for the U.S. Preventive Services Task Force (Internet, Evidence Syntheses, No. 139. Table 1, Statin Dosing and ACC/AHA Classification of Intensity). Rockville, MD: Agency for Healthcare Research and Quality. Available at: https://www.ncbi.nlm.nih.gov/books/NBK396417/table/ch1.t1/27905702

[bibr13-02698811211060307] Collins R Reith C Emberson J , et al. (2016) Interpretation of the evidence for the efficacy and safety of statin therapy. The Lancet 388(10059): 2532–2561. 10.1016/S0140-6736(16)31357-5 27616593

[bibr14-02698811211060307] Cooper CM Godlewska B Sharpley AL , et al. (2018) Interferon-α induces negative biases in emotional processing in patients with hepatitis C virus infection: A preliminary study. Psychological Medicine 48(6): 998–1007. 28889805 10.1017/S0033291717002379 PMC5767463

[bibr15-02698811211060307] Corsini A Bellosta S Baetta R , et al. (1999) New insights into the pharmacodynamic and pharmacokinetic properties of statins. Pharmacology & Therapeutics 84(3): 413–428. 10665838 10.1016/s0163-7258(99)00045-5

[bibr16-02698811211060307] De Giorgi R De Crescenzo F Cowen PJ (2021c) Effects of various statins on depressive symptoms: Is there enough evidence? Journal of Affective Disorders 295: 1093–1094. 34706419 10.1016/j.jad.2021.09.009

[bibr17-02698811211060307] De Giorgi R De Crescenzo F Rizzo Pesci N , et al. (2021b) Statins for major depressive disorder: A systematic review and meta-analysis of randomized controlled trials. PLoS ONE 16(3): e0249409. 10.1371/journal.pone.0249409 PMC8009386 33784356

[bibr18-02698811211060307] De Giorgi R Rizzo Pesci N Quinton A , et al. (2021a) Statins in depression: An evidence-based overview of mechanisms and clinical studies. Frontiers in Psychiatry 12: 702617. 34385939 10.3389/fpsyt.2021.702617 PMC8353114

[bibr19-02698811211060307] Deakin JFW Graeff FG (1991) 5-HT and mechanisms of defence. Journal of Psychopharmacology 5(4): 305–315. 22282829 10.1177/026988119100500414

[bibr20-02698811211060307] Enache D Pariante CM Mondelli V (2019) Markers of central inflammation in major depressive disorder: A systematic review and meta-analysis of studies examining cerebrospinal fluid, positron emission tomography and post-mortem brain tissue. Brain, Behavior, and Immunity 81: 24–40. 31195092 10.1016/j.bbi.2019.06.015

[bibr21-02698811211060307] Endo A Kuroda M Tanzawa K (1976) Competitive inhibition of 3-hydroxy-3-methylglutaryl coenzyme A reductase by ML-236A and ML-236B fungal metabolites, having hypocholesterolemic activity. FEBS Letters 72(2): 323–326. 16386050 10.1016/0014-5793(76)80996-9

[bibr22-02698811211060307] Eysenck SBG Eysenck HJ Barrett P (1985) A revised version of the Psychoticism Scale. Personality and Individual Differences 6: 21–29.

[bibr23-02698811211060307] Faul F Erdfelder E Lang AG , et al. (2007) G*Power 3: A flexible statistical power analysis program for the social, behavioral, and biomedical sciences. Behavior Research Methods 39(2): 175–191. 17695343 10.3758/bf03193146

[bibr24-02698811211060307] Felger J Treadway M (2017) Inflammation effects on motivation and motor activity: Role of dopamine. Neuropsychopharmacol 42: 216–241. 10.1038/npp.2016.143 PMC5143486 27480574

[bibr25-02698811211060307] First MB Williams JBW Karg RS , et al. (2016) User’s Guide for the SCID-5-CV Structured Clinical Interview for DSM-5® Disorders: Clinical Version. Washington, DC: American Psychiatric Publishing.

[bibr26-02698811211060307] Godlewska BR Harmer CJ (2021) Cognitive neuropsychological theory of antidepressant action: A modern-day approach to depression and its treatment. Psychopharmacology 238: 1265–1278. 31938879 10.1007/s00213-019-05448-0 PMC8062380

[bibr27-02698811211060307] Grier JB (1971) Nonparametric indexes for sensitivity and bias: Computing formulas. Psychological Bulletin 75(6): 424–429. 5580548 10.1037/h0031246

[bibr28-02698811211060307] Gulbins E Palmada M Reichel M , et al. (2013) Acid sphingomyelinase-ceramide system mediates effects of antidepressant drugs. Nature Medicine 19(7): 934–938. 10.1038/nm.3214 23770692

[bibr29-02698811211060307] Haghighi M Khodakarami S Jahangard L , et al. (2014) In a randomized, double-blind clinical trial, adjuvant atorvastatin improved symptoms of depression and blood lipid values in patients suffering from severe major depressive disorder. Journal of Psychiatric Research 58: 109–114. 25130678 10.1016/j.jpsychires.2014.07.018

[bibr30-02698811211060307] Harmer CJ Cowen PJ (2013) ‘It’s the way that you look at it’: A cognitive neuropsychological account of SSRI action in depression. Philosophical Transactions of the Royal Society B 368: 20120407. 10.1098/rstb.2012.0407 PMC3638386 23440467

[bibr31-02698811211060307] Harmer CJ Bhagwagar Z Perrett DI , et al. (2003) Acute SSRI administration affects the processing of social cues in healthy volunteers. Neuropsychopharmacology 28(1): 148–152. 12496951 10.1038/sj.npp.1300004

[bibr32-02698811211060307] Harmer CJ Cowen PJ Goodwin GM (2011) Efficacy markers in depression. Journal of Psychopharmacology 25(9): 1148–1158. 20530590 10.1177/0269881110367722

[bibr33-02698811211060307] Harmer CJ Duman RS Cowen PJ (2017) How do antidepressants work? New perspectives for refining future treatment approaches. The Lancet Psychiatry 4(5): 409–418. 28153641 10.1016/S2215-0366(17)30015-9 PMC5410405

[bibr34-02698811211060307] Harmer CJ O’Sullivan U Favaron E , et al. (2009) Effect of acute antidepressant administration on negative affective bias in depressed patients. The American Journal of Psychiatry 166(10): 1178–1184. 19755572 10.1176/appi.ajp.2009.09020149

[bibr35-02698811211060307] Harmer CJ Shelley NC Cowen PJ , et al. (2004) Increased positive versus negative affective perception and memory in healthy volunteers following selective serotonin and norepinephrine reuptake inhibition. The American Journal of Psychiatry 161(7): 1256–1263. 15229059 10.1176/appi.ajp.161.7.1256

[bibr36-02698811211060307] Horder J Browning M Di Simplicio M , et al. (2012) Effects of 7 days of treatment with the cannabinoid type 1 receptor antagonist, rimonabant, on emotional processing. Journal of Psychopharmacology 26(1): 125–132. 21406493 10.1177/0269881111400649

[bibr37-02698811211060307] Hudgins LC Parker TS Levine DM , et al. (2003) A single intravenous dose of endotoxin rapidly alters serum lipoproteins and lipid transfer proteins in normal volunteers. Journal of Lipid Research 44(8): 1489–1498. 12754273 10.1194/jlr.M200440-JLR200

[bibr38-02698811211060307] Husain MI Chaudhry IB Khoso AB , et al. (2019) Adjunctive simvastatin for treatment-resistant depression: Study protocol of a 12-week randomised controlled trial. BJPsych Open 5(1): e13. 10.1192/bjo.2018.84 PMC6381416 30762508

[bibr39-02698811211060307] Husain MI Chaudhry IB Khoso AB , et al. (2020) Minocycline and celecoxib as adjunctive treatments for bipolar depression: A multicentre, factorial design randomised controlled trial. The Lancet Psychiatry 7(6): 515–527. 32445690 10.1016/S2215-0366(20)30138-3

[bibr40-02698811211060307] IBM (2020) IBM SPSS Statistics for Macintosh (Version 27.0, Released 2020). Armonk, NY: IBM.

[bibr41-02698811211060307] Jain MK Ridker PM (2005) Anti-inflammatory effects of statins: Clinical evidence and basic mechanisms. Nature Reviews Drug Discovery 4(12): 977–987 16341063 10.1038/nrd1901

[bibr42-02698811211060307] Köhler-Forsberg O Otte C Gold SM , et al. (2020) Statins in the treatment of depression: Hype or hope? Pharmacology & Therapeutics 215: 107625. 32652185 10.1016/j.pharmthera.2020.107625

[bibr43-02698811211060307] Köhler O Gasse C Petersen L , et al. (2016) The effect of concomitant treatment with SSRIs and statins: A population-based study. The American Journal of Psychiatry 173(8): 807–815. 27138586 10.1176/appi.ajp.2016.15040463

[bibr44-02698811211060307] Law MR Wald NJ Rudnicka AR (2003) Quantifying effect of statins on low density lipoprotein cholesterol, ischaemic heart disease, and stroke: Systematic review and meta-analysis. The BMJ 326: 1423. 12829554 10.1136/bmj.326.7404.1423 PMC162260

[bibr45-02698811211060307] Lee LH Shui G Farooqui AA , et al. (2009) Lipidomic analyses of the mouse brain after antidepressant treatment: Evidence for endogenous release of long-chain fatty acids? The International Journal of Neuropsychopharmacology 12(7): 953–964. 19203412 10.1017/S146114570900995X

[bibr46-02698811211060307] Lee MC Peng TR Chen BL , et al. (2021) Effects of various statins on depressive symptoms: A network meta-analysis. Journal of Affective Disorders 293: 205–213. 34217957 10.1016/j.jad.2021.06.034

[bibr47-02698811211060307] Lundqvist ED Flykt A Öhman A (1998) The Karolinska Directed Emotional Faces (KDEF, CD ROM). Stockholm: Psychology Section, Department of Clinical Neuroscience, Karolinska Institutet.

[bibr48-02698811211060307] McFarland AJ Anoopkumar-Dukie S Arora DS , et al. (2014) Molecular mechanisms underlying the effects of statins in the central nervous system. International Journal of Molecular Sciences 15(11): 20607–20637. 25391045 10.3390/ijms151120607 PMC4264186

[bibr49-02698811211060307] Macin SM Perna ER Farías EF , et al. (2005) Atorvastatin has an important acute anti-inflammatory effect in patients with acute coronary syndrome: Results of a randomized, double-blind, placebo-controlled study. American Heart Journal 149(3): 451–457. 15864233 10.1016/j.ahj.2004.07.041

[bibr50-02698811211060307] McIntyre RS Subramaniapillai M Lee Y , et al. (2019) Efficacy of adjunctive infliximab vs placebo in the treatment of adults with bipolar I/II depression: A randomized clinical trial. JAMA Psychiatry 76(8): 783–790. 31066887 10.1001/jamapsychiatry.2019.0779 PMC6506894

[bibr51-02698811211060307] Miller AH Haroon E Felger JC , et al. (2017) Therapeutic implications of brain-immune interactions: Treatment in translation. Neuropsychopharmacology 42(1): 334–359. 27555382 10.1038/npp.2016.167 PMC5143492

[bibr52-02698811211060307] Mogg K Bradley BP (2002) Selective orienting of attention to masked threat faces in social anxiety. Behaviour Research and Therapy 40: 1403–1414. 12457635 10.1016/s0005-7967(02)00017-7

[bibr53-02698811211060307] Molero Y Cipriani A Larsson H , et al. (2020) Associations between statin use and suicidality, depression, anxiety, and seizures: A Swedish total-population cohort study. The Lancet Psychiatry 7(11): 982–990. 33069320 10.1016/S2215-0366(20)30311-4 PMC7606915

[bibr54-02698811211060307] Okudan N Belviranli M (2020) High dose simvastatin and rosuvastatin impair cognitive abilities of healthy rats via decreasing hippocampal neurotrophins and irisin. Brain Research Bulletin 165: 81–89. 33010350 10.1016/j.brainresbull.2020.09.019

[bibr55-02698811211060307] Otte C Chae WR Nowacki J , et al. (2020) Simvastatin add-on to escitalopram in patients with comorbid obesity and major depression (SIMCODE): Study protocol of a multicentre, randomised, double-blind, placebo-controlled trial. BMJ Open 10: e040119. 10.1136/bmjopen-2020-040119 PMC7709515 33262189

[bibr56-02698811211060307] Paine NJ Bosch JA Ring C , et al. (2015) Induced mild systemic inflammation is associated with impaired ability to improve cognitive task performance by practice. Psychophysiology 52(3): 333–341. 25366393 10.1111/psyp.12360

[bibr57-02698811211060307] Parsaik AK Singh B Murad MH , et al. (2014) Statins use and risk of depression: A systematic review and meta-analysis. Journal of Affective Disorders 160: 62–67. 24370264 10.1016/j.jad.2013.11.026

[bibr58-02698811211060307] Pessiglione M Seymour B Flandin G , et al. (2006) Dopamine-dependent prediction errors underpin reward-seeking behaviour in humans. Nature 442(7106): 1042–1045. 16929307 10.1038/nature05051 PMC2636869

[bibr59-02698811211060307] Pringle A Browning M Cowen PJ , et al. (2011) A cognitive neuropsychological model of antidepressant drug action. Progress in Neuro-Psychopharmacology & Biological Psychiatry 35(7): 1586–1592. 20673783 10.1016/j.pnpbp.2010.07.022

[bibr60-02698811211060307] Raison CL Rutherford RE Woolwine BJ , et al. (2013) A randomized controlled trial of the tumor necrosis factor antagonist infliximab for treatment-resistant depression: The role of baseline inflammatory biomarkers. JAMA Psychiatry 70(1): 31–41. 22945416 10.1001/2013.jamapsychiatry.4 PMC4015348

[bibr61-02698811211060307] Redlich C Berk M Williams LJ , et al. (2014) Statin use and risk of depression: A Swedish National Cohort Study. BMC Psychiatry 14: 348. 25471121 10.1186/s12888-014-0348-y PMC4266881

[bibr62-02698811211060307] Rey A (1964) L’examen clinique en psychologic [The Clinical Examination in Psychology]. Paris: Universitaires de France.

[bibr63-02698811211060307] Salagre E Fernandes BS Dodd S , et al. (2016) Statins for the treatment of depression: A meta-analysis of randomized, double-blind, placebo-controlled trials. Journal of Affective Disorders 200: 235–242. 27148902 10.1016/j.jad.2016.04.047

[bibr64-02698811211060307] Salvadore G Nash A Bleys C , et al. (2018) A double-blind, placebo-controlled, multicenter study of sirukumab as adjunctive treatment to a monoaminergic antidepressant in adults with major depressive disorder. In: American College of Neuropsychopharmacology annual meeting, 9–13 December, Hollywood, FL.

[bibr65-02698811211060307] Schneider M Levant B Reichel M , et al. (2017) Lipids in psychiatric disorders and preventive medicine. Neuroscience & Biobehavioral Reviews 76: 336–362. 27317860 10.1016/j.neubiorev.2016.06.002

[bibr66-02698811211060307] Schultz BG Patten DK Berlau DJ (2018) The role of statins in both cognitive impairment and protection against dementia: A tale of two mechanisms. Translational Neurodegeneration 7: 5. 29507718 10.1186/s40035-018-0110-3 PMC5830056

[bibr67-02698811211060307] Sizar O Khare S Jamil RT , et al. (2021) Statin Medications. Treasure Island, FL: StatPearls Publishing. Available at: https://www.ncbi.nlm.nih.gov/books/NBK430940/28613690

[bibr68-02698811211060307] Snaith RP Hamilton M Morley S , et al. (1995) A scale for the assessment of hedonic tone the Snaith-Hamilton Pleasure Scale. The British Journal of Psychiatry 167(1): 99–103. 7551619 10.1192/bjp.167.1.99

[bibr69-02698811211060307] Spielberger CD Gorsuch RL Lushene RD (1970) STAI Manual. Palo Alto, CA: Consulting Psychologists Press.

[bibr70-02698811211060307] Van Diepen JA Berbée JF Havekes LM , et al. (2013) Interactions between inflammation and lipid metabolism: Relevance for efficacy of anti-inflammatory drugs in the treatment of atherosclerosis. Atherosclerosis 228(2): 306–315. 23518178 10.1016/j.atherosclerosis.2013.02.028

[bibr71-02698811211060307] Walsh AEL Browning M Drevets WC , et al. (2018) Dissociable temporal effects of bupropion on behavioural measures of emotional and reward processing in depression. Philosophical Transactions of The Royal Society B Biological Sciences 373(1742): 20170030. 10.1098/rstb.2017.0030 PMC5790828 29352029

[bibr72-02698811211060307] Walther A Cannistraci CV Simons K , et al. (2018) Lipidomics in major depressive disorder. Frontiers in Psychiatry 9: 459. 30374314 10.3389/fpsyt.2018.00459 PMC6196281

[bibr73-02698811211060307] Watson D Clarke LA Tellegen A (1988) Development and validation of brief measures of positive and negative affect: The positive and negative affect schedule scales. Journal of Personality and Social Psychology 54: 1063–1070. 3397865 10.1037//0022-3514.54.6.1063

[bibr74-02698811211060307] Young AW Rowland D Calder AJ , et al. (1997) Facial expression megamix: Tests of dimensional and category accounts of emotion recognition. Cognition 63: 271–313. 9265872 10.1016/s0010-0277(97)00003-6

[bibr75-02698811211060307] Young-Xu Y Chan KA Liao JK , et al. (2003) Long-term statin use and psychological well-being. Journal of the American College of Cardiology 42(4): 690–697 12932603 10.1016/S0735-1097(03)00785-X PMC2673913

[bibr76-02698811211060307] Zangara A Blair RJ Curran HV (2002) A comparison of the effects of a beta-adrenergic blocker and a benzodiazepine upon the recognition of human facial expressions. Psychopharmacology 163: 36–41. 12185398 10.1007/s00213-002-1120-4

