# Supplementary material for: The effects of atorvastatin on emotional processing, reward learning, verbal memory and inflammation in healthy volunteers: An experimental medicine study
Source: J Psychopharmacol. 2021 Dec 6;35(12):1479–87. doi: 10.1177/02698811211060307 (PMC8652357; doi:10.1177/02698811211060307)

**SUPPLEMENTARY MATERIAL**

**S1 - Sample size calculation**
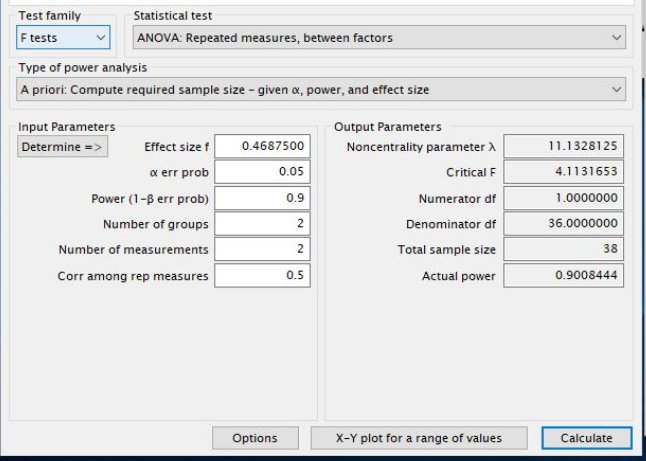


**S2 - Descriptive statistics for Bond-Lader Visual Analog Scale (BL- VAS)**

**BL-VAS**

**
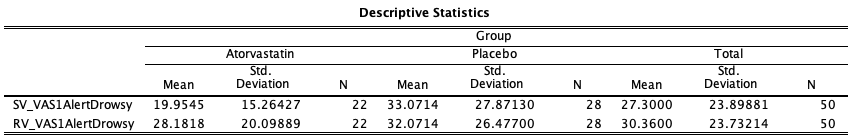
**

**
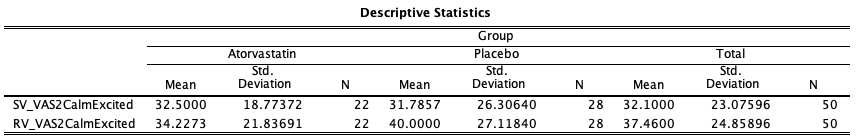
**

**
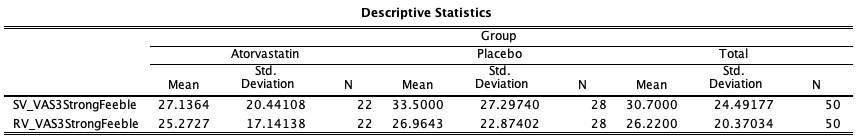
**

**
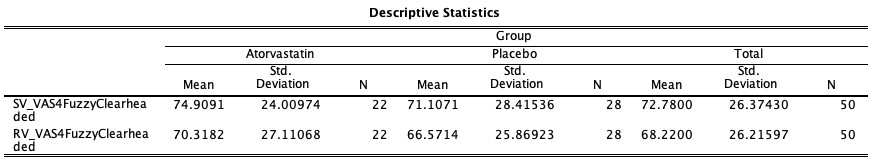
**

**
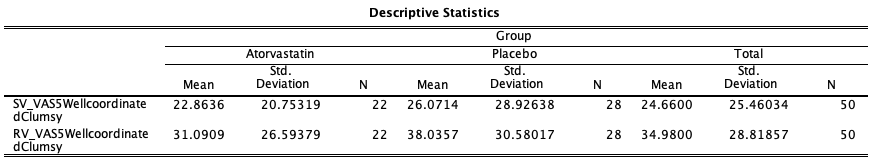
**

**
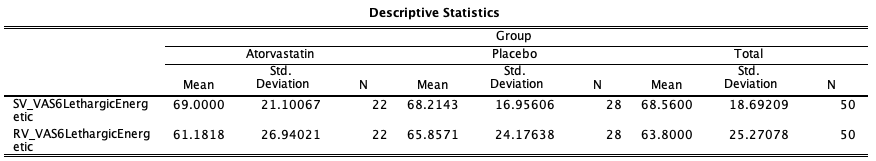
**

**
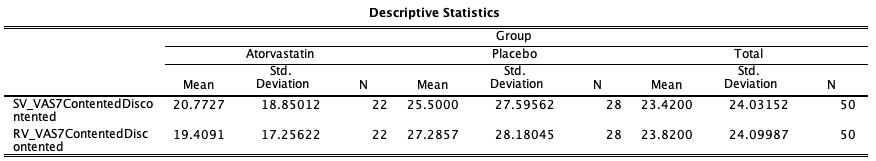
**

**
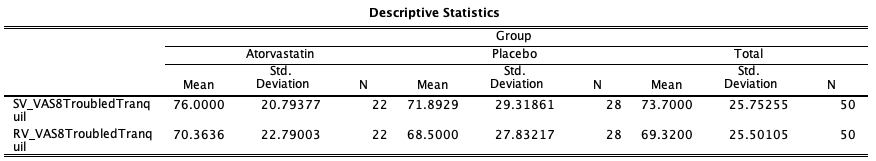
**

**
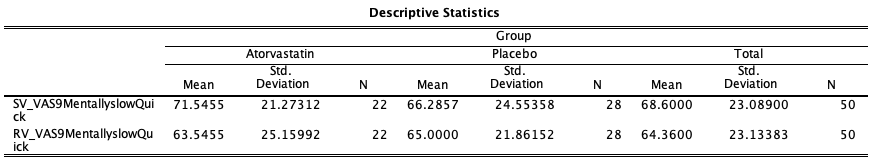
** **
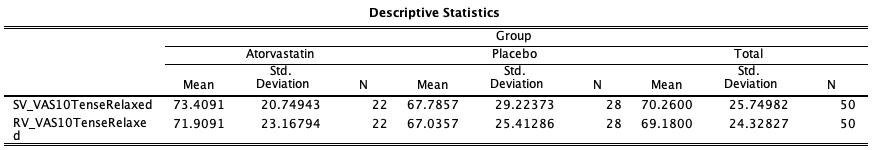
**
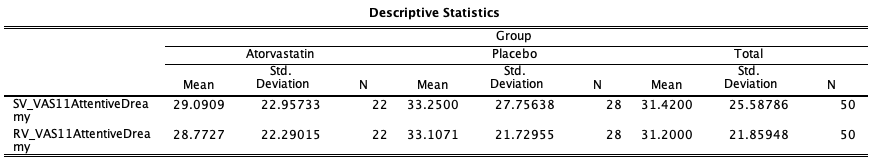

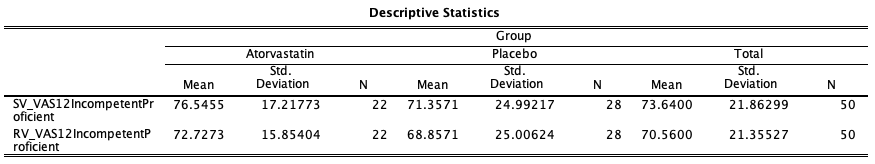


**
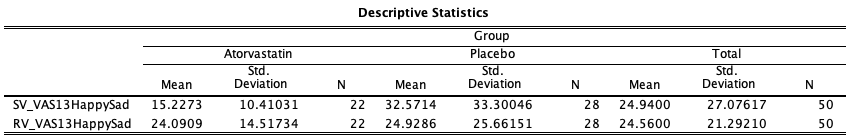
**

**
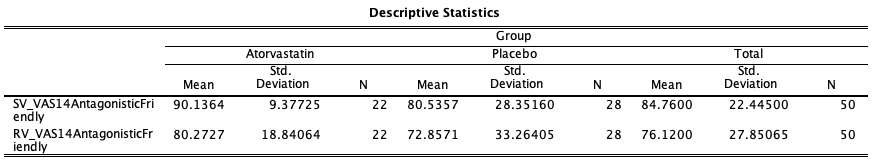
**

**
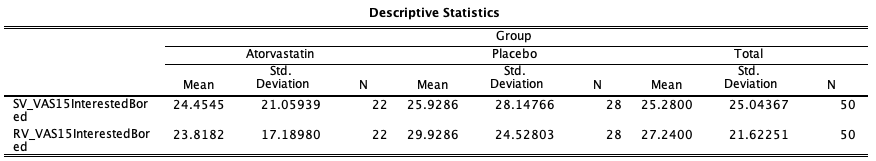
** **
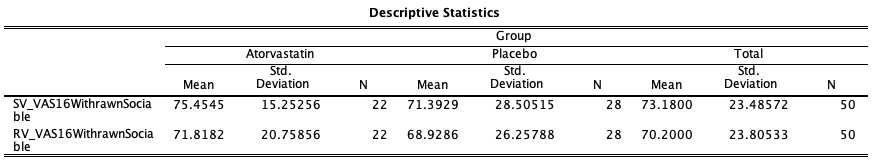
**

**S3 - Descriptive statistics for behavioural tasks**

Outcomes measured are defined in the Methods.

***FERT***

FERT accuracy, all emotions


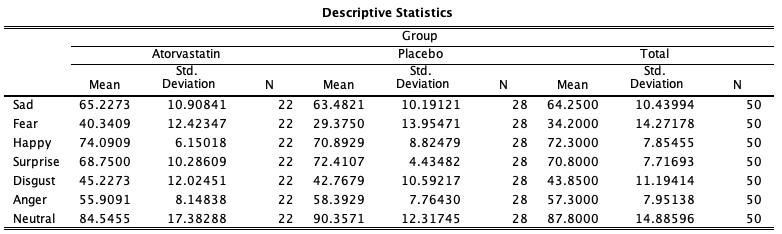


FERT accuracy, positive vs negative emotions


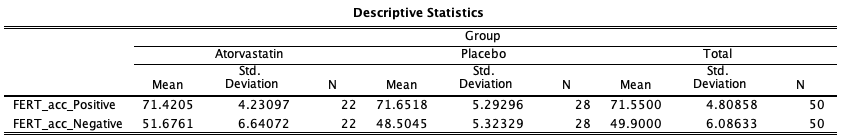


FERT reaction times, all emotions


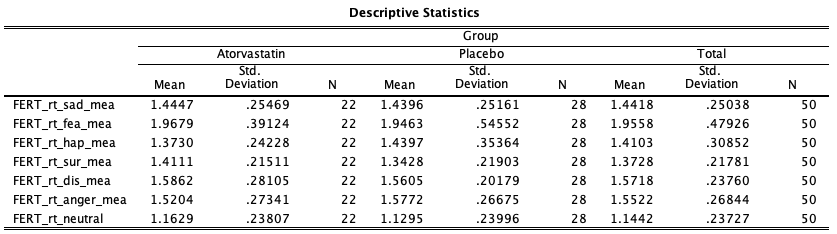


FERT reaction times, positive vs negative emotions


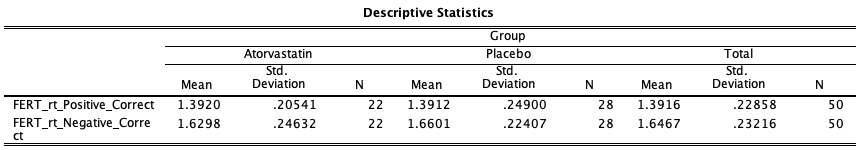


FERT misclassifications, all emotions


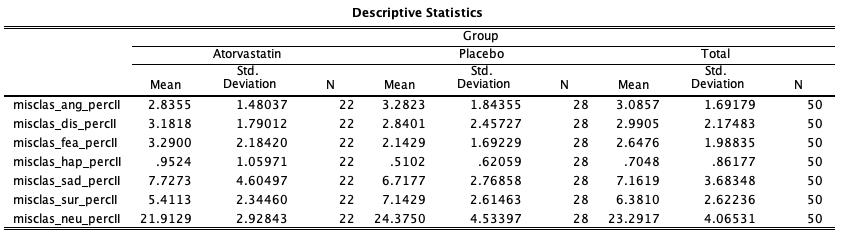


FERT sensitivity index (d’), all emotions


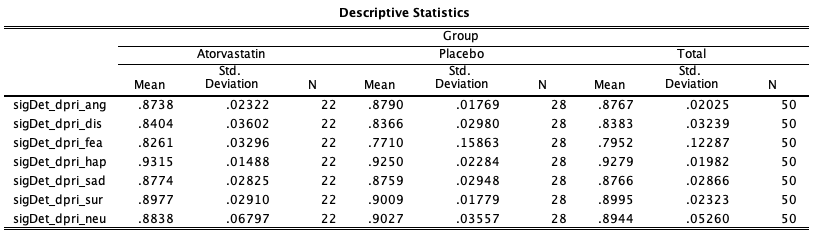


FERT response bias (beta), all emotions


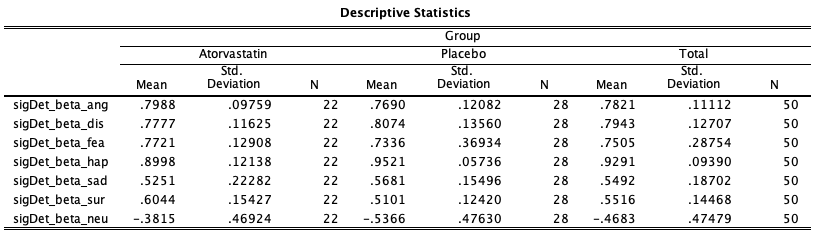


***ECAT***

ECAT, accuracy


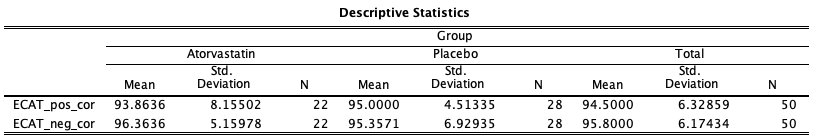


ECAT, reaction times


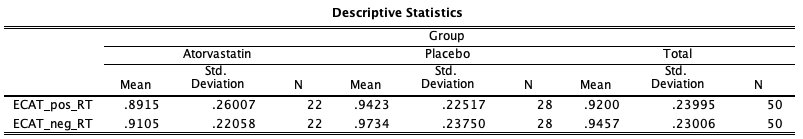


***EREC***

EREC, correctly recalled words


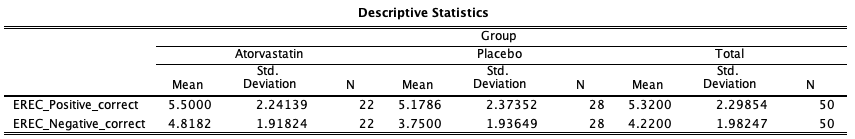


EREC, incorrectly recalled words


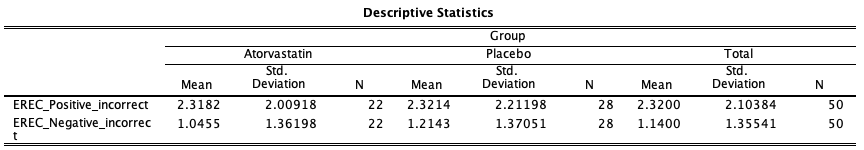


***EMEM***

EMEM, accuracy


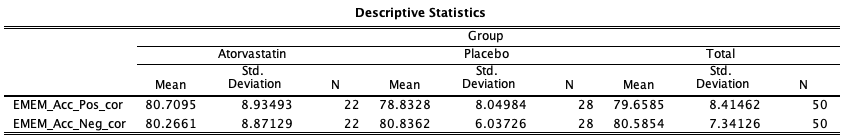


EMEM, reaction times


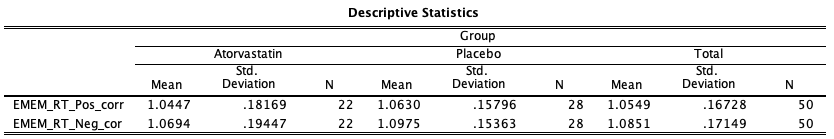


EMEM, misclassifications


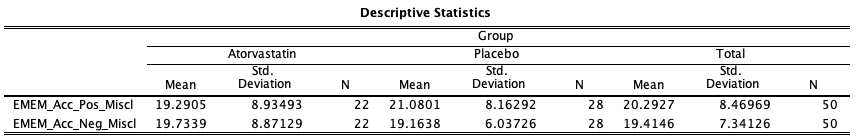


***FDOT***

FDOT, attentional vigilance


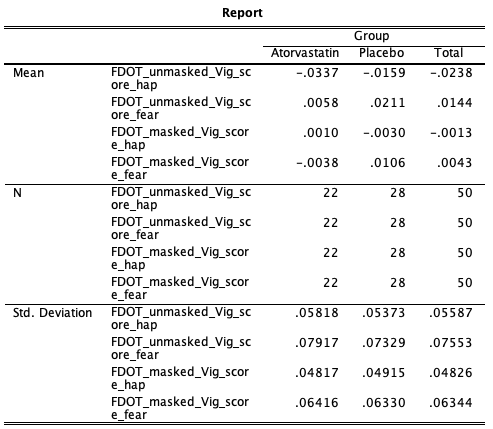


***PILT***

PILT, amount won/lost


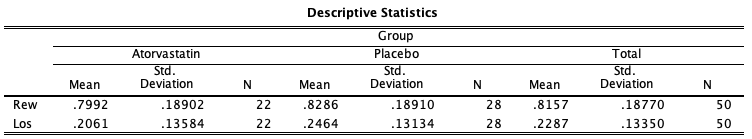


PILT, number of choice switches


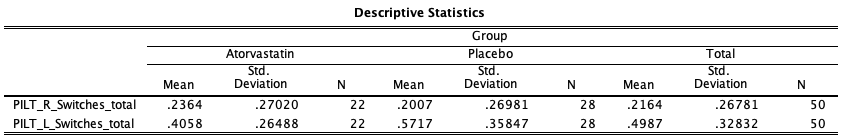


***AVLT***

AVLT, total correct


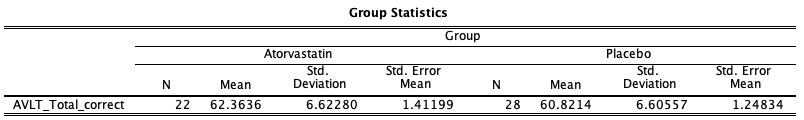


AVLT, short delay, long delay, recognition


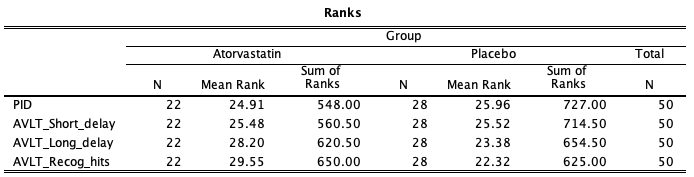

Supplement: sj-docx-1-jop-10.1177_02698811211060307 – Supplemental material for The effects of atorvastatin on emotional processing, reward learning, verbal memory and inflammation in healthy volunteers: An experimental medicine study [file sj-docx-1-jop-10.1177_02698811211060307.docx]
